# Understanding the Promotion of Health Equity at the Local Level Requires Far More than Quantitative Analyses of Yes-No Survey Data: Comment on "Health Promotion at Local Level in Norway: The Use of Public Health Coordinators and Health Overviews to Promote Fair Distribution Among Social Groups"

**DOI:** 10.15171/ijhpm.2018.70

**Published:** 2018-07-31

**Authors:** Dennis Raphael

**Affiliations:** School of Health Policy and Management, York University, Toronto, ON, Canada.

**Keywords:** Norway, Health Equity, Health Promotion, Naturalistic Inquiry

## Abstract

Health promotion is a complex activity that requires analytic methods that recognize the contested nature of it definition, the barriers and supports for such activities, and its embeddedness within the politics of distribution. In this commentary I critique a recent study of municipalities’ implementation of the Norwegian Public Health Act that employed analysis of "yes" or "no" responses from a large survey. I suggest the complexity of health promotion activities can be best captured through qualitative methods employing open-ended questions and thematic analysis of responses. To illustrate the limitations of the study, I provide details of how these methods were employed to study local public health unit (PHU) activity promoting health equity in Ontario, Canada.

## Introduction

Human behaviour is complicated, and this is especially the case when dealing with issues of social justice, fairness and equity.^1^ Add to this mix contested issues of fair distribution and health promotion, a dab of social structure, social process, and politics, and we have the recipe for a very complex situation that calls for critical analysis that involve far more than quantitative analysis of yes-no survey data.^2^ This appears to be especially the case when we consider attempts by the State to promote health equity across a nation through municipal governance.

In the article *Health Promotion at the Local Level in Norway: The Use of Public Health Coordinators and Health Overviews to Promote Fair Distribution among Social Groups* we have such a situation.^3^ How do local authorities work to achieve health equity by creating health overviews, employing a public health coordinator (PHC), and working with local politicians and civil society organizations? The issue is even more complicated by the terms fair distribution, health promotion, and health equity being contested with a clear consensus of their meaning difficult to achieve. And with these issues interacting with politics – *Who Gets What, When, and How?*^4^ – specific analytic methods are required to make sense of what is happening on the ground to achieve health equity across Norway.

The authors recognize the complexity of these areas and apply complex logistic regression models to tease out which factors may be responsible for achieving “fair distribution among social groups in political decision-making” and “fair distribution among social groups in local health promotion initiatives.” Unfortunately, the data fed into these analyses are “yes” and “no” responses to survey questions without inquiry into the nature of these interventions nor the respondents’ understandings of these terms. The result is rather little insight into the structures and processes necessary to implement health equity by municipal authorities. There is implicit recognition of these problems by the authors as they devote over three pages of the article to their discussion of findings. To my mind, the investigation of these issues requires moving beyond statistical analyses of simple survey data through traditional statistical analyses to methods that can describe and make sense of the real life complexity of promoting health equity through health promotion activity.

Inquiry into complex situations of intersecting factors and systems of factors by traditional research methods led to the backlash against positivist notions of social reality and the methods commonly employed to explain this reality.^5^ The development of what has been termed naturalistic inquiry, qualitative research methods, ethnographic research, as well as a host of other interpretive approaches were developed to understand these complexities. Researchers attempting to understand how municipalities are meeting or not meeting various objectives of the Norwegian Public Health Act should consider the shortcomings and advantages of these differing research approaches.

## The Study

Regarding the article under consideration, the first problem is the possibility that respondents had very different understandings of the terms and issues raised in the survey questions. The second is that promoting health, especially at the municipal level involves complicated processes involving local politics, ideological beliefs of stakeholders, and the ability of governments to effect significant changes in the distribution of economic and social resources amongst their residents.

As noted, respondents simply answered “yes” or “no” to a series of what some might consider straightforward questions but are anything but. The questions that were asked are supplemented with what I would have explored through open-ended questions.

“*Does the municipality have a PHC?”*

What is the mandate of the PHC? What background training and skills does the PHC have? What decision-making ability does the PHC have?

“*Has the municipality developed an overview of inhabitants’ health status, and the positive and negative determinants of health?”*

What are the understandings held by staff and stakeholders of the meaning of health and health status and the positive and negative determinants of health? What are the micro-level behavioural factors, meso-level community factors or macro-level structural factors that constitute health and the determinants of health? How do these factors interact? What are the goals of the health overview?

*The first variable was whether the municipality had “strengthened the competence base for health promotion. *”

*How do you and other stakeholders conceive of health promotion? What skills do you and they believe are included in this skill set?*

*The second variable was whether the municipality had “increased collaboration with voluntary organizations.*

“*Does the municipality collaborate with external actors in health promotion networks?”*

“*Has the municipality established cross-sectorial working groups for health promotion at the strategic level?”*

What was the nature of these collaborations? What are their goals? Who took on leadership roles? How do you understand the terms health equity, health promotion, and fair distribution?

“*Are considerations of fair distribution a priority in political decision-making?”*

How do you and stakeholders conceive the meaning of fair distribution? What are areas where local actions are possible? What are the barriers and supports to such efforts? What are some examples of decisions that were taken or not taken?

“*Are considerations of fair distribution a priority in the area of local health promotion initiatives?”*

What are the connections between local health promotion and fair distribution? How are these translated into action?

## A Personal Example

The best way to gain an understanding of these complex processes is through open-ended theoretically-driven inquiry. We had the opportunity to investigate similar issues related to how local public health units (PHUs) in Ontario, Canada conceived of the social determinants of health (SDOH) and the PHU’s role in improving their quality and equitable distribution.^6-8^ We focused on nine PHUs and interviewed nine medical officers of health and their key staff persons with a set of open-ended questions that included among others (Full set of questions available from author):

### Thinking about the Social Determinants of Health

1. How do you think about the term “social determinants of health”?
2. How have you come to think about it this way?

### Unit Activities

1. What activities has the unit carried out to address the SDOH?
2. What unit structures have been established to support these activities?

### Local Supports and Barriers

1. What are some of the features of the local community and its institutions that have facilitated action by the unit on the SDOH?
2. What are some of the features about the local community and its institutions that have created barriers to action by the unit on the SDOH?

### Political Environments

1. Is there a Board of Health that is responsible for overseeing PHU activities?
2. What role does it play in PHU activities?

### Moving Forward

1. How do you think about the future role of PHUs in Ontario in addressing the SDOH?
2. What could be done to facilitate these activities?

**Figure 1:**
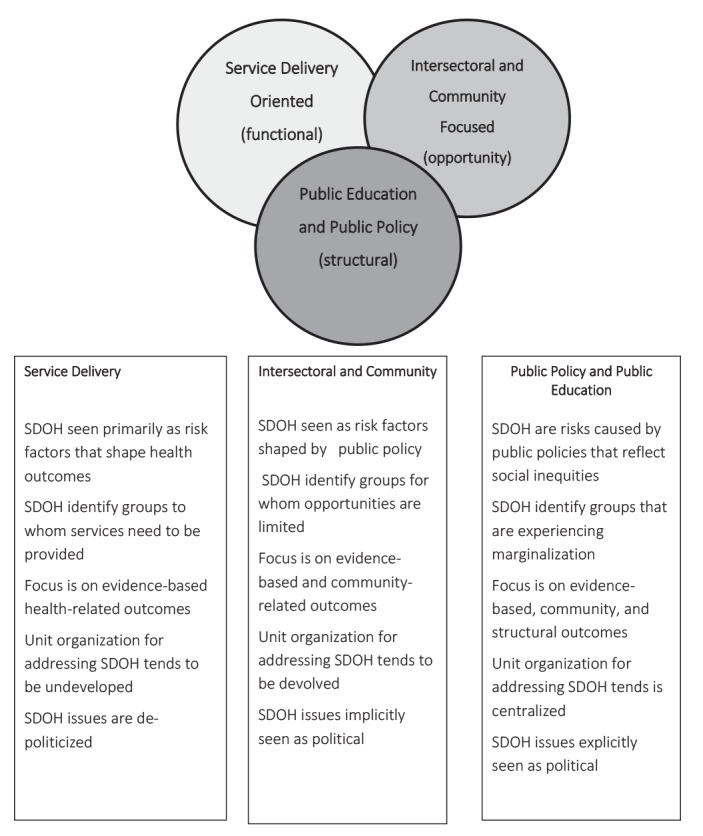
The Empirical: Public Health Units’ (PHUs’) Differing Approaches to Addressing the Social Determinants of Health (SDOH).

We then carried out thematic analyses of their responses and collected documents and reports related to these activities. We were struck by the complexity of the factors that contributed to different forms of activities amongst these nine units. There were profound differences in understandings of the definition of SDOH, the role that PHUs should play in responding to them, as well as complex contextual factors that shaped how local units went about their activities. Figure 1 presents the three general approaches we identified for addressing these issues.

*Service delivery-oriented* PHUs limited themselves to service-related activities that responded to SDOH-related needs of clients while PHUs identified as *Intersectoral and Community-Focused* carried out both service-delivery and community-based intersectoral activities designed to improve services and stimulate health-promoting public policy. *Public Policy/Public Education* PHUs also carried out service delivery and engaged in policy-related community-based activities, but additionally assumed a leadership role in carrying out public policy advocacy and public education about the SDOH. Certainly, such differences would be present among the many Norwegian municipalities attempting to promote health equity.

**Figure 2:**
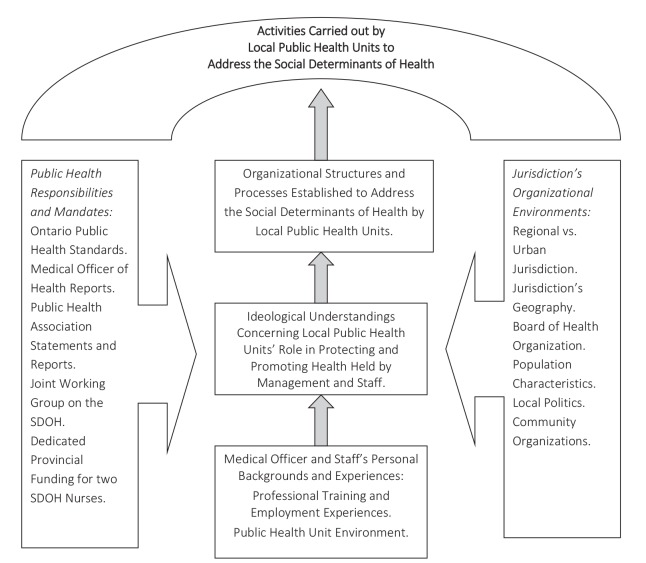
The Real: Potential Structures and Processes as well as Influences Upon Local Public Health Units (PHUs) Approaches to Addressing the Social Determinants of Health (SODH).

To make sense of these differences, we employed a critical realist perspective which identifies the real, actual, and empirical levels of these phenomena (see Figure 2). The *real* is the explication of the societal structures and powers that have the capacity to allow a phenomenon to occur. For example, like the Norwegian Public Health Act, the Ontario Public Health Standards mandate addressing the SDOH and Ontario’s Medical Officer of Health has reported on the need to address the SDOH in two Annual Reports. In addition, like the Norway situation, the Ministry of Health and Long-Term Care provides dedicated funding for two staff for each of the 36 PHUs to address the SDOH.

The *actual* refers to whether these structures and powers are activated such that a PHU carries out local SDOH actions. The actual therefore considers how the jurisdiction’s organizational environments and characteristics, the PHU’s working environment, and the training and priorities of lead staff members either activate or inhibit these powers. In Norway, this would be the various factors that contributed to or blocked health equity-related activities by municipalities. These issues were not considered in the Norway article.

The empirical is what the PHU does to address the SDOH; that is, its specific programs, activities, and initiatives. In Norway, this would be the activities into which the survey inquired but provided no details about. The value of the critical realist approach is that it reveals that powers may exist unexercised, such that what is happening does not preclude what can happen. We propose applying these qualitative approaches towards understanding issues related to the implementation of the Norwegian Public Health Act.

### Depth Versus Breadth

One critique of the qualitative approach is its inability to gather data from the breadth of potential respondents. In the study we carried out, we limited ourselves to nine PHUs. However, in another study that examined implementation of a health equity-focused video animation we were able to gather rich data from 18 units that employed an open-ended survey that inquired into understandings, goals, and experiences related to local health equity activities.^9^ Such forms of inquiry can be applied to gather rich data from a very larger number of respondents. While all of Norway’s 428 municipalities could not be included in such a study, sampling in the range of 75-100 municipalities or so could provide data that could be generalized to the entire population.

### The Norwegian Versus the Ontario Scene

There are some differences between the welfare regimes and legislative environments of the nation of Norway and the province of Ontario. In Norway promoting health equity is clearly on the national and local health agendas while in Ontario such activity has not been explicitly placed on the public policy agenda. However, this does not mitigate the importance of inquiry into health promoters’ understandings of these concepts as even in Norway there may be significant “lifestyle drift” in health promoters’ and municipal authorities’ actual activities.^10^

It should also be noted that the Ontario study involved medical officers of health and senior/key public health staff while the Hagen et al. study collected data from PHCs who were often part-time and in lower-level positions. If anything, this difference affirms the need to understand these PHCs’ understandings of health equity and health promotion concepts and how these understandings are translated into local action.

## Conclusion

The authors suggest that insights from this study could be complemented by qualitative inquiry using a mixed-methods approach. I doubt whether such an add-on by itself would justify the effort extended in the quantitative study. The study reported could not capture the complexity inherent in promoting fair distribution for health equity by municipalities. It provided good baseline data as to how many municipalities hired PHCs and their participation in various activities but little else. Independent of the analyses reported, the authors provide a thoughtful analysis of the role that PHCs and municipalities can play in promoting health equity. However, findings related to temporal sequencing of hiring PHCs provided little insight into these findings as municipalities may have many different reasons for not having hired PHCs. Most importantly these PHCs and the municipal authorities with whom they work may have different understandings as to the meaning of health promotion and health equity as well as fair distribution. It did not provide insight into the different assumptions PHCs may have held about what is expected of them. Such inquiry into these understandings seems essential in promoting Norwegian attempts to promote health equity for all.

## Ethical issues

Not applicable.

## Competing interests

Author declares that he has no competing interests.

## Author’s contribution

DR is the single author of the paper.
